# Allostatic load index in patients with pituitary tumours: a case control study

**DOI:** 10.3389/fendo.2025.1676246

**Published:** 2025-10-29

**Authors:** Martyna Strzelec, Dorota Szcześniak, Iga Zendran-Zahorska, Justyna Kuliczkowska-Płaksej, Natalia Słoka, Krzysztof Kujawa, Marek Bolanowski, Aleksandra Jawiarczyk-Przybyłowska

**Affiliations:** ^1^ Department and Clinic of Endocrinology and Internal Medicine, Wroclaw Medical University, Wroclaw, Poland; ^2^ Department and Clinic of Psychiatry, Wroclaw Medical University, Wroclaw, Poland; ^3^ Statistical Analysis Centre, Wroclaw Medical University, Wroclaw, Poland

**Keywords:** allostatic load, allostasis, pituitary tumour, acromegaly, stress

## Abstract

**Introduction:**

Prolonged exposure to pathogenic stress factors leads to multisystemic consumption of the body and adverse changes resulting in the development of allostatic load (AL). Stress plays a crucial role in the pathophysiology of many diseases, including endocrinopathies. The purpose of our study was to evaluate the allostatic load index in patients with pituitary tumours and compare it to a control group.

**Materials and methods:**

The study group included 58 patients with hormonally active pituitary tumours and 52 patients without pituitary dysfunction, representing the control group. The AL index (ALI) was calculated based on 16 parameters grouped into the following categories: anthropometric parameters, cardiovascular markers, lipid and carbohydrate metabolism parameters, and inflammatory and hormonal markers.

**Results:**

In the group of patients with pituitary tumours, a statistically significantly higher AL index was noticed, regardless of the endocrine function of the adenoma, compared to the control group [7.00 (5.00–9.00) vs. 3.50 (2.00–5.00), p < 0.001]. Age significantly affected the AL index, while no such relationship was observed for education. Analysing specific AL biomarkers, patients with pituitary tumours had significantly higher Body Mass Index (BMI), systolic blood pressure and diastolic blood pressure. Individuals in the study group showed significantly higher levels of insulin, triglycerides and interleukin 6, and significantly lower levels of high-density lipoprotein cholesterol, dehydroepiandrosterone sulphate and albumin, compared to the control group.

**Conclusions:**

The results of our study indicate the usefulness of the AL index as an integrated tool for assessing the cumulative impact of stress factors in pituitary diseases. In addition, patients with hormonally active pituitary tumours presented a higher cardio-metabolic risk. It is necessary to analyse the clinimetric data affecting AL, which is the next step of our study.

## 1 Introduction

Allostasis can be defined as the process of constantly maintaining stability in the internal environment of an organism, under conditions of constant variability of the external environment, which is perceived by the individual as stressful. The assumption of much greater variability and flexibility of the internal environment is a distinguishing feature from the previously described concept of homeostasis (1). Brain activity is involved in the process of allostasis, by recognising and evaluating stressors, and initiating responses to them, through autonomic and neuroendocrine mechanisms (2).

The cumulative effects of an allostatic state result in the development of allostatic load (AL), defined as the occurrence of adverse changes in the body in response to the chronic effects of multiple stressors over a lifetime, including developmental experiences, genetic predisposition, and environmental, psychosocial and lifestyle factors (3). AL involves dysregulation in various stress systems, including the immune system, the hypothalamic-pituitary-adrenal (HPA) axis, the autonomic nervous system and general proteomic or metabolomic pathways (4, 5). This process affects health and leads to multisystem wear and tear on the brain and body (6–8). If the imbalance persists for an extended period, even while adequate energy reserves are maintained, the body begins to show symptoms of allostatic overload (AO) (9).

The allostatic load index (AL index) is used to assess AL, which has been calculated based on clinical norms, and through the distribution of markers in a control group or clinical population (10). The knowledge of the mechanisms of AL has made it possible to identify several biomarkers, which include: anthropometric parameters [body weight, Body Mass Index (BMI), Waist-Hip Ratio (WHR)], cardiovascular [heart rate (HR), systolic blood pressure (SBP), diastolic blood pressure (DBP)], metabolic [total cholesterol (TC), high-density lipoprotein cholesterol (HDL cholesterol), low-density lipoprotein cholesterol (LDL cholesterol), triglycerides (TG), insulin, glucose, glycated haemoglobin (HbA1c), creatinine], neuroendocrine [cortisol, dehydroepiandrosterone sulphate (DHEA-S), epinephrine, norepinephrine], and immune-inflammatory [fibrinogen, C-reactive protein (CRP), albumin, interleukin 6 (IL-6)]. In addition, the functional status and quality of life of patients in various disease entities are evaluated as a measure of clinimetric methods (11, 12).

Studies have confirmed the association between higher AL and changes in various brain areas, especially in elderly people [hippocampus, white matter volume, cerebral grey matter volume and density], patients with schizophrenia spectrum disorders [cortex, vault, hippocampus and choroid plexus], and overweight people [cerebral white matter pathways, cerebral cortex grey matter volume and cerebral cortex thickness] (13). The multisystem dysregulation that characterises AL can lead to cellular damage and degenerative diseases, particularly in the older population (14), and during critical periods of brain development (childhood, adolescence), which are characterised by increased neuroplasticity and increased sensitivity to epigenetic effects, AL can exert long-term effects on individual neural networks, leading to permanent neuroendocrine changes (15). High AL is associated with an increased risk of all-cause mortality and cardiovascular mortality, making it a valuable prognostic indicator for patient outcomes (16). Studies have shown that higher AL scores predict higher coronary heart disease (CHD) risk, supporting the hypothesis that cumulative biological dysregulation may act as an early determinant of atherosclerosis and CHD (17). AL is also linked to various cancer-related outcomes, including cancer-specific stress, tumour pathology, and cancer-specific mortality. A one-unit increase in AL is associated with a 9% increased risk of cancer-specific mortality, indicating its potential as a screening tool for high-risk individuals (5). Individuals with multiple sclerosis were also studied, and it was found that patients in this group had significantly higher AL compared to healthy controls (18). Furthermore, stress-related psychiatric disorders, such as depression and anxiety, were also associated with increased AL (6). In patients suffering from schizophrenia and in a first episode of psychosis, higher AL has been correlated with cognitive decline (19, 20). In addition, post-traumatic stress disorder (PTSD) has also been linked to increased AL in women who have experienced sexual abuse (21).

The concept of allostatic load is crucial to understanding the development and progression of endocrine disorders. When adaptive systems (neuronal, neuroendocrine and immune mechanisms) are over-stimulated or fail to shut down properly, this leads to physiological dysregulation, which can manifest itself through fluctuations in various biomarkers, including cortisol, DHEA-S, and catecholamines (such as norepinephrine and epinephrine) (5, 22). The mechanisms of this hormonal dysregulation are often not explained by traditional medical assessments. The effect of fluctuations in the levels of certain hormones, such as increased cortisol levels and lower DHEA-S levels, has been linked to the metabolic syndrome and other endocrine disorders (15). Another issue is chronic stress and allostatic load contributing to the onset and progression of endocrine diseases. Psychological and psychiatric symptoms are common in both the prodromal and active phases of these diseases, and residual symptoms may persist even after treatment (23, 24). The inclusion of allostatic burden assessment through clinical measurements and biomarkers can provide a more comprehensive understanding of a patient’s psychosocial environment and its impact on endocrine health (25). Studies analysing the importance of the AL concept among patients with pituitary tumours are lacking. In this study, we compared the AL index and physiological and biochemical markers between the study group – patients with pituitary tumours and healthy control individuals in the context of assessing AL.

## 2 Materials and methods

### 2.1 Participants

**Figure 1:**
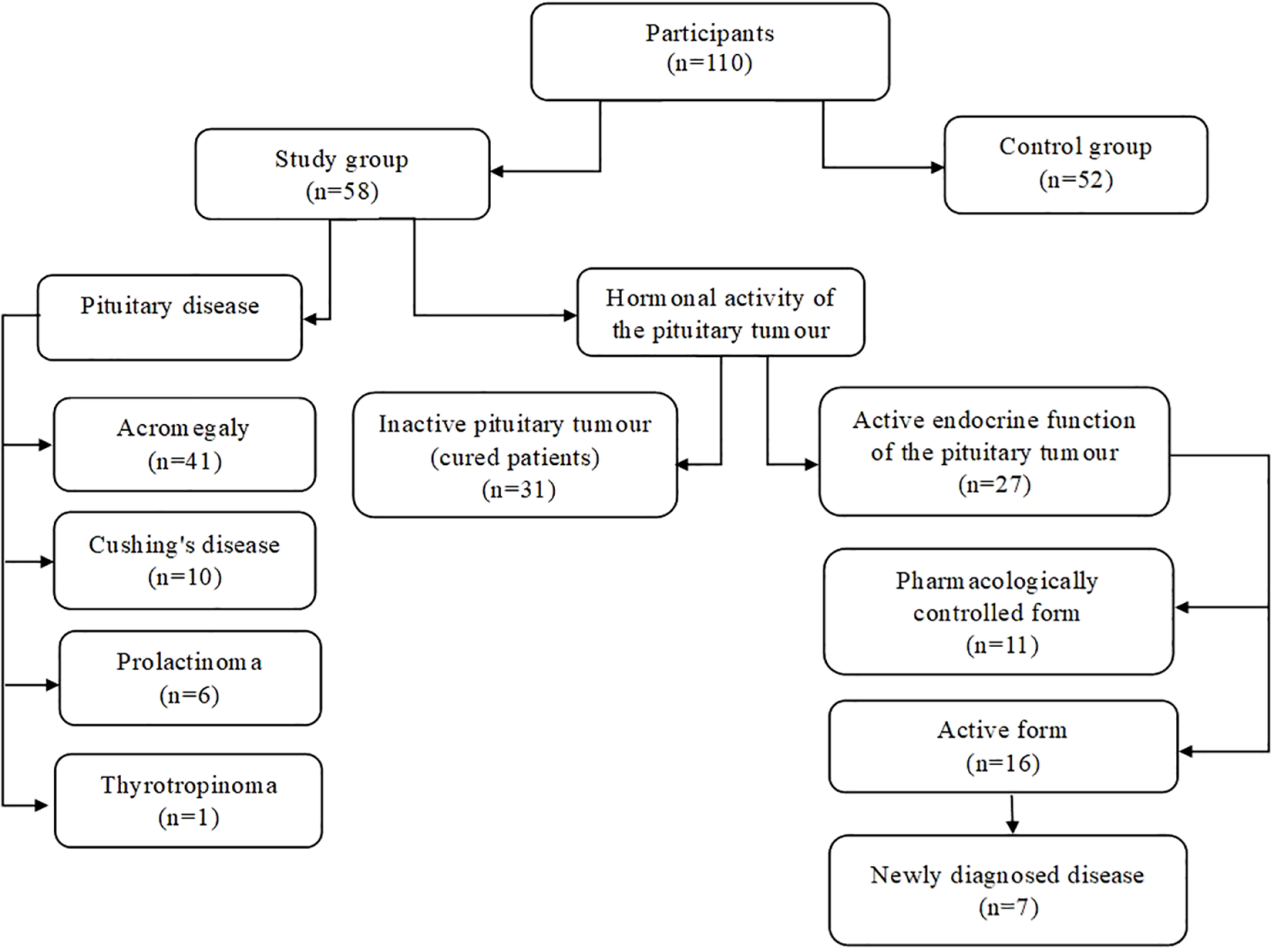
Division of study participants.

Participants in this study were patients hospitalised at the Department of Endocrinology and Internal Medicine in Wroclaw, Poland. We collected data from January 2024 to February 2025. The study group consisted of 58 patients with hormonally active pituitary tumours (36 females and 22 males, mean age 50.95 years) (**Figure 1**). The criteria for inclusion in the study group were age over 18 years and current or past diagnosis of pituitary disease (acromegaly, Cushing’s disease, prolactinoma, thyrotropinoma), based on current guidelines and recommendations for the primary disease.

The study group included: 41 patients with acromegaly, 10 with Cushing’s disease, 6 prolactinomas and 1 case of thyrotropinoma were diagnosed. The criteria for the diagnosis of acromegaly, according to the 14th Acromegaly Consensus Conference, were insulin-like growth factor 1 (IGF-I) values above 1.3 times the upper limit of normal (ULN) for age, and characteristic clinical signs of the disease (26). Patients with ACTH-dependent Cushing’s syndrome fulfilled the diagnostic criteria for the disease (abnormal circadian rhythm with late-night salivary cortisol levels, impaired glucocorticoid feedback with an overnight 1 mg dexamethasone suppression test or a 2-day low-dose dexamethasone test and increased bioavailable cortisol with 24-hour urinary free cortisol, and increased or normal level of ACTH—adrenocorticotropic hormone) and had confirmed pituitary adenoma localisation on Magnetic Resonance Imaging (MRI) (27). The diagnosis of prolactinoma and thyrotropinoma was based on clinical symptoms, a constellation of hormonal findings [consecutively abnormal prolactin diurnal profile, hyperthyroxinemia with unsuppressed thyroid-stimulating hormone (TSH) levels] and finding a tumour on pituitary imaging (28, 29). Among the participants in the study group were 7 patients with newly diagnosed disease. We divided the whole study group into 2 subgroups according to the hormonal activity of the pituitary tumour. Twenty-seven patients were classified into the group with active endocrine function of the pituitary tumour (16 patients with the active form of the disease and 11 with the pharmacologically controlled form), while 31 patients were included in the group with an inactive pituitary tumour (cured patients).

The exclusion criteria for the control group were pituitary tumours confirmed by MRI or pituitary secretion disorders. The control group included 52 subjects (31 females and 21 males, mean age 47.02 years) without pituitary dysfunction, age- and gender-matched to patients with pituitary tumours.

The study was conducted according to the guidelines of the Declaration of Helsinki, and the Ethics Committee of the Wroclaw Medical University, Poland, approved the study protocol (number 162/2024). All participants provided written informed consent prior to their inclusion in this study.

### 2.2 Clinical assessment

The interview conducted with the study participants included questions on sociodemographic data, such as age, sex, education level and place of residence. In addition, the interview included clinical data: medical history—duration of illness, method of treatment, medications taken, pituitary MRI results, comorbidities, and family history—and was extended by an analysis of the current and previous medical records. Patients’ anthropometric data were recorded: body weight (kg), height (cm) and BMI (kg/m2), and blood pressure (mm/Hg) and heart rate (bpm) measurements were taken.

### 2.3 Biochemical parameters

Fasting venous blood samples were collected from all study participants. The levels of the following markers necessary for calculating the allostatic load index were analysed: total cholesterol, LDL cholesterol, HDL cholesterol, TG, glucose, insulin, HbA1c, CRP, albumin, IL-6, cortisol, and DHEA-S. Atherogenicity indices were calculated, including Castelli index 1 and 2, plasma atherogenic index (API) and atherogenic coefficient (AC). Insulin resistance indices [homeostatic model assessment of insulin resistance (HOMA-IR) and quantitative insulin sensitivity check index (QUICKI)] were also estimated. In addition, hormone levels were examined: growth hormone (GH), IGF-I, insulin-like growth factor binding protein (IGFBP-3), follicle-stimulating hormone (FSH), luteinising hormone (LH), testosterone, estradiol, TSH, free triiodothyronine (fT3), free thyroxine (fT4), ACTH, prolactin (PRL), and sex hormone binding globulin (SHBG). Creatinine, estimated glomerular filtration rate (eGFR), haemoglobin, vitamin D, uric acid, and N-terminal prohormone of brain natriuretic peptide (NT-proBNP) levels were also analysed.

### 2.4 AL index

We calculated the AL index according to the method described (30), based on 16 parameters grouped into the following categories: 1) cardiovascular markers: systolic and diastolic blood pressure, and resting heart rate; 2) anthropometric measurements: body mass index (BMI); 3) lipid metabolism parameters: total cholesterol, LDL cholesterol, HDL cholesterol and TG; 4) parameters of carbohydrate metabolism: fasting glucose, insulin and HbA1c; 5) inflammatory markers: CRP, albumin and IL-6; and 6) hormonal parameters: cortisol and DHEA-S.

Every marker with a value above the standard accepted by the performing laboratory (in the case of laboratory results) was scored ‘1’. The exceptions to this rule were HDL cholesterol, albumin and DHEA-S, for which ‘1’ point was assigned when the value was below the accepted norms. Then, the sum of all markers scored as ‘1’ point in each category (cardiovascular markers, anthropometric measurements, lipid metabolism parameters, carbohydrate metabolism parameters, inflammatory markers and hormonal parameters) was divided by the total number of markers in each category to ensure that each biological system contributed equally to the final AL index score.

To account for the effect of pharmacotherapy used for comorbidities, maximum points were assigned to the lipid metabolism parameters (if dyslipidemia was treated), carbohydrate metabolism parameters (if diabetes or prediabetes was treated), and SBP and DBP (if hypotensive drugs were used). The total AL index (ALI) was calculated as the sum of the scores in each category.

### 2.5 Statistics

The normality of the data in the groups was checked using the Shapiro-Wilk test (**Supplementary Table S1**, **Supplementary Table S2**). For categorical data, the Pearson’s Chi-square test of independence was used (X^2^ value is presented). The Student’s t-test with Welch’s correction was used (t value is presented) to compare the groups with a normal data distribution (age, fat (%)), and if the distribution was non-normal, the Mann-Whitney U test (M-W test) was used (with W value presented). Variance homogeneity (when using the t-test) was checked using Levene’s test (in all cases, p > 0.250). Three model types were used to assess the effect of the group, age and education on the AL indexes, depending on the explained variable type:

- A linear regression model was used to examine the effect of age and education on the total score AL index as it was considered a numeric variable (R function: lm (ALI_total ~ Group + Age + Education, data);
- A binary logistic regression was used in the case of anthropometric AL as this variable is binary (0/1) (R function: glm (ALI_anthrop ~ Group + Age + Education, family = ‘binomial’, data);
- A Poisson regression was used for cardiovascular, lipid metabolism, carbohydrate metabolism, neuroendocrine, and inflammatory AL index, as they considered count variables (R function: glm (ALI cardiovascular ~ Group + Age + Education |Group, data, dist = ‘poisson’).

The assumption of linear relationships between the log-odds of and age in the above model b) was checked using the Box-Tidwell test (the effect of the interaction age * log(age) was statistically insignificant at p = 0.78). Due to the large number of zero values in the AL index variables, the zero-inflated Poisson (ZIP) models were also considered, using the AIC to compare the model performance, and the Vuong test, (non nested likelihood ratio test) which checks the test’s distinguishability (R function: nonnest (m_zip, m_pois), where: m_zip—results of ZIP, m_pois—results of Poisson regression). Based on this test and the AIC, the results of the two models were not distinguishable (**Supplementary Table S3**), so the second one (Poisson regression, simpler) was finally used. Theeducation was considered an factorialvariable with the elementary school as the reference level. The description statistics and the tests of the differences between groups were done using Statistica (version 13.3). All regression types were done in R, using the packages: ‘stat’ (for linear, logistic, and Poisson regression), ‘pscl’ (for zero-inflated-Poisson regression), and ‘nonnest2’ (for model distinguishability). The Breusch-Pagan test (R package ‘lmtest’) was used to check for residual heteroskedasticity, and GVIF of coefficients checked for collinearity among predictors (function ‘vif’ from the R package ‘car’). The level of significance was set at p < 0.05.

## 3 Results

**Table 1** presents the general characteristics of the patients with pituitary tumours and controls. The level of education was significantly different between the groups (p < 0.001). The control group was dominated by higher education (86.54%). The education of patients with pituitary tumours was more varied, with occupational and primary education present, which was absent in the controls. A significant difference was also observed in the place of residence. Individuals in the study group were more frequently living in a city from 50, 000 to 150, 000 inhabitants, while participants in the control group were more commonly from a city with over 500, 000 inhabitants. Patients with pituitary tumours also had significantly higher body weight (p < 0.001) compared to the control group. Analysing the prevalence of civilization chronic diseases, we noted that nicotinism (p = 0.008), hypertension (p < 0.001), insulin resistance (p < 0.001), pre-diabetes (p < 0.001) and diabetes (p = 0.047) were significantly more frequent in the study group compared to the controls.

**Table 1:** Characteristics of the study (patients with pituitary tumours) and control groups.

Parameters	Sample size (Study group; control group)	Study group (n = 58)	Control group (n = 52)	Test value	*p*-value
Sex (F %/M %)	58; 52	62.07/37.93	59.62/40.38	0.07	0.792^c^
Age (years)	58; 52	50.95 ± 14.42	47.02 ± 13.29	1.49	0.140^w^
Education n (%):	58; 52			30.62	**< 0.001** ^c^
Primary		1 (1.72)	0 (0.00)		
Vocational		11 (18.97)	0 (0.00)		
Secondary		25 (43.10)	7 (13.46)		
Higher		21 (36.21)	45 (86.54)		
Place of residence n (%):	58; 52			11.38	**0.023** ^c^
Village		17 (29.31)	13 (25.00)		
City up to 50, 000		16 (27.59)	15 (28.85)		
City from 50, 000 to 150, 000		11 (18.97)	1 (1.92)		
City from 150, 000 to 500, 000		0 (0.00)	1 (1.92)		
City over 500, 000		14 (24.14)	22 (42.31)		
Weight (kg)	58; 52	83.00 (73.00–98.00)	69.50 (62.00–82.00)	2203	**< 0.001** ^m^
Height (cm)	58; 52	170.00 (164.00–176.00)	172.00 (163.50–176.50)	−0.11	0.909^w^
Fat (%)	21; 27	33.46 ± 7.90	31.67 ± 6.05	0.86	0.395^w^
Nicotinism n (%)	57; 51	19 (33.33)	6 (11.76)	7.04	**0.008** ^c^
Family history of cardiology n (%):	55; 51			0.47	0.495^c^
Positive		15 (27.27)	11 (21.57)		
Negative		40 (72.73)	40 (78.43)		
Arterial hypertension n (%)	58; 52	34 (58.62)	9 (17.31)	19.65	**< 0.001** ^c^
Hypercholesterolemia n (%)	58; 52	32 (55.17)	25 (48.08)	0.55	0.457^c^
Insulin resistance n (%)	52; 50	19 (36.54)	3 (6.00)	14.05	**< 0.001** ^c^
Prediabetic state n (%)	58; 50	18 (31.03)	1 (2.00)	15.61	**< 0.001** ^c^
Diabetes mellitus n (%)	58; 51	11 (18.97)	1 (1.96)	8.01	**0.047** ^c^
Illness duration (years)	58; -	8.86 (2.00–13.00)			
Pituitary tumour size at time of diagnosis n (%):	56; -				
Microadenoma		13 (23.21)			
Macroadenoma		43 (76.79)			
Max tumour size (cm)	44; -	1.50 (0.98–2.25)			
Disease activity n (%):	58; -				
Active		16 (27.59)			
Controlled		11 (18.97)			
Cured		31 (53.45)			
Treatment n (%):					
Surgical	58; -	47 (81.03)			
Pharmacotherapy	55; -	16 (29.09)			
Radiotherapy	58; -	9 (15.52)			
Hypopituitarism n (%)	58; -	14 (24.14)			
Hypogonadism n (%)	55; -	15 (27.27)			

study group, control group, ^W^—Welch’s t-test; ^c^—Chi-square test; ^m^—Mann–Whitney test; x ± SD/Me (Q1–Q3); x ± SD—mean and standard deviation; Me (Q1–Q3)—median and quartiles; *p*—statistical significance. F, female; M, male; BMI, body mass index; HR, heart rate; SBP, systolic blood pressure; DBP, diastolic blood pressure. Bold values - statistical significance, p < 0.05

**Figure 2:**
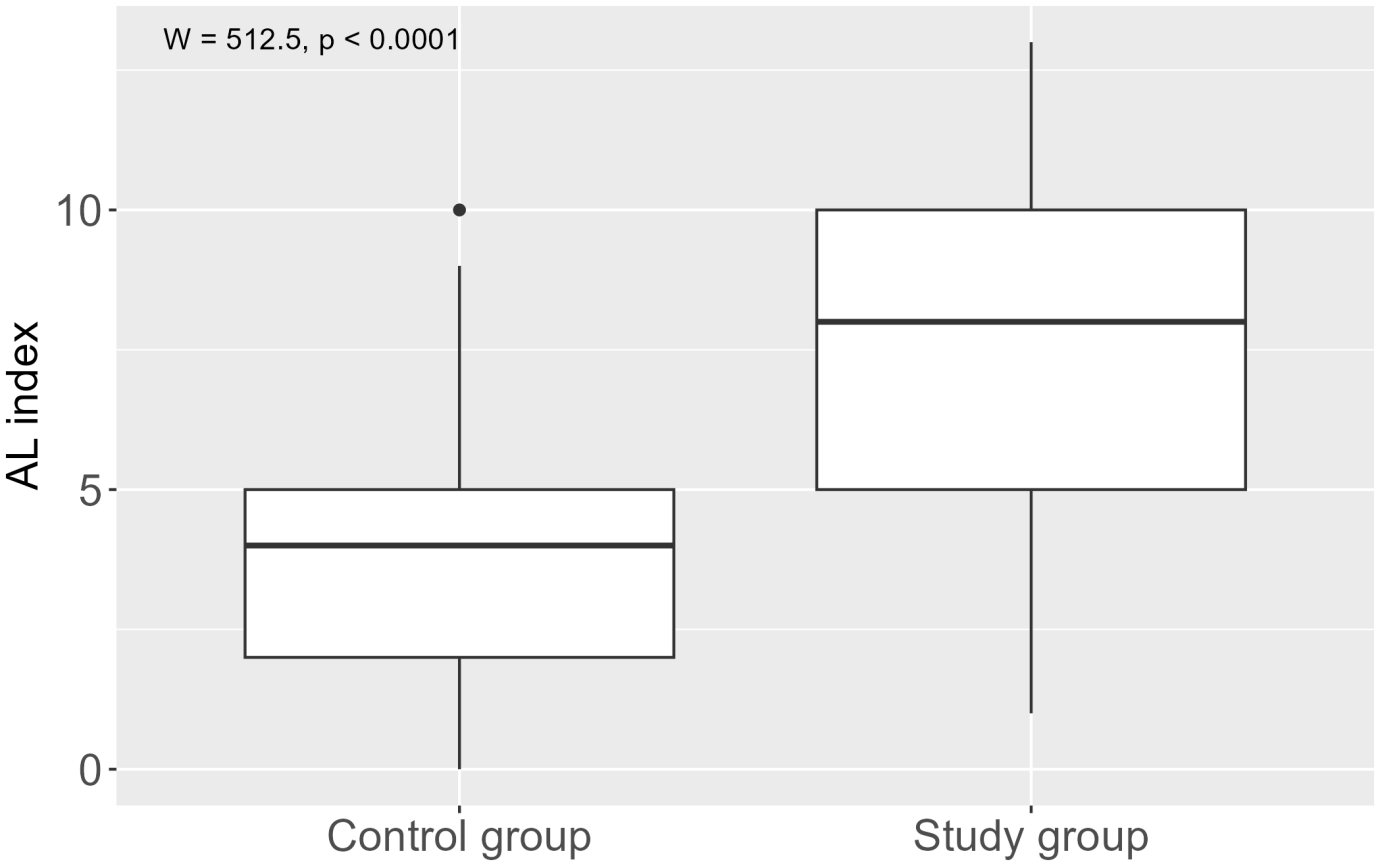
Allostatic load index in pituitary tumour patients and control group.

Patients with pituitary tumours had a significantly higher AL total index compared to the control group [7.00 (5.00–9.00) vs. 3.50 (2.00–5.00), M-W test: p < 0.001] (**Figure 2**). After the IGF-I levels were taken into account in the calculation of the total AL index, the value of the aforementioned index was found to be significantly higher in both the patients in the entire study group [8.00 (5.00–10.00) vs. 4.00 (2.00–5.00), M-W test: p < 0.001] and the patients with acromegaly [7.50 (5.00–9.25) vs. 4.00 (2.00–5.00), M-W test: p < 0.001], compared to the control group. The predominant components of the AL index in the study group were parameters of lipid metabolism (31.9%), cardiovascular parameters (20.9%) and inflammatory parameters (14.5%), while parameters of lipid metabolism (37.8%), and inflammatory (16.1%) and neuroendocrine (15.6%) components predominated in the control group. Age significantly influenced the AL index (p < 0.001), while no such association was observed for education. **Table 2** shows a comparison of the components of the AL index and the effect of age and education on these components.

**Table 2:** Effect of age and education on the components of the AL index in the study group.

AL Index	Variable	p-value	Effect size	95% CI	
			OR/RR	Low	High
Anthropometric (OR)	Group (Study)	**0.006**	4.06	1.49	11.09
	Age	0.317	1.02	0.98	1.06
	technical	0.991	na		
	high school	0.991	na		
	university	0.991	na		
Cardiovascular (RR)	Group (Study)	**< 0.001**	2.59	1.61	4.17
	Age	**0.015**	1.02	1.00	1.03
	technical	0.991	na		
	high school	0.991	na		
	university	0.991	na		
Lipid metabolism (RR)	Group (Study)	0.232	1.22	0.84	1.76
	Age	**< 0.001**	1.03	1.01	1.04
	technical	0.991	na		
	high school	0.991	na		
	university	0.991	na		
Carbohydrate metabolism (RR)	Group (Study)	**0.002**	3.45	1.49	8.00
	Age	**< 0.001**	1.04	1.01	1.06
	technical	0.991	na		
	high school	0.991	na		
	university	0.991	na		
Neuroendocrine (RR)	Group (Study)	0.048	1.67	1.01	2.77
	Age	0.850	1.00	0.98	1.02
	technical	0.946	1.07	0.12	9.46
	high school	0.956	0.93	0.12	7.43
	university	0.939	1.07	0.14	8.29
Inflammatory (RR)	Group (Study)	0.090	1.54	0.94	2.52
	Age	0.501	1.01	0.99	1.02
	technical	0.994	na		
	high school	0.994	na		
	university	0.994	na		

p, statistical significance; OR, Odds Ratio; RR, Rate Ratio; CI, Confidence Interval; na, not available. Elementary education not presented as reference level.

Logistic regression used for anthropometric AL index, and Poisson regression for all others cases. Bold values - statistical significance, p < 0.05

There were significantly higher values for the anthropometric (p = 0.006), cardiovascular (p < 0.001) and carbohydrate metabolism (p < 0.001) components in the study group compared to the control group. In terms of lipid, neuroendocrine and inflammatory metabolism, we found no significant differences between groups.

After including the IGF-I levels in the calculation of the AL index, the comparative analysis conducted showed significantly higher levels of the neuroendocrine component of the AL index in the study group (p = 0.002), including the subgroup with acromegaly (p < 0.001), compared to the control group. The highest values of the neuroendocrine component of the AL index were observed in the group of patients with acromegaly.

A significant effect of age on the cardiovascular (RR = 1.02; p = 0.015), lipid metabolism (RR = 1.03; p < 0.001) and carbohydrate metabolism (RR = 1.04; p < 0.001) components was observed. Educational level had no significant effect on any of the analysed components.

**Figure 3:**
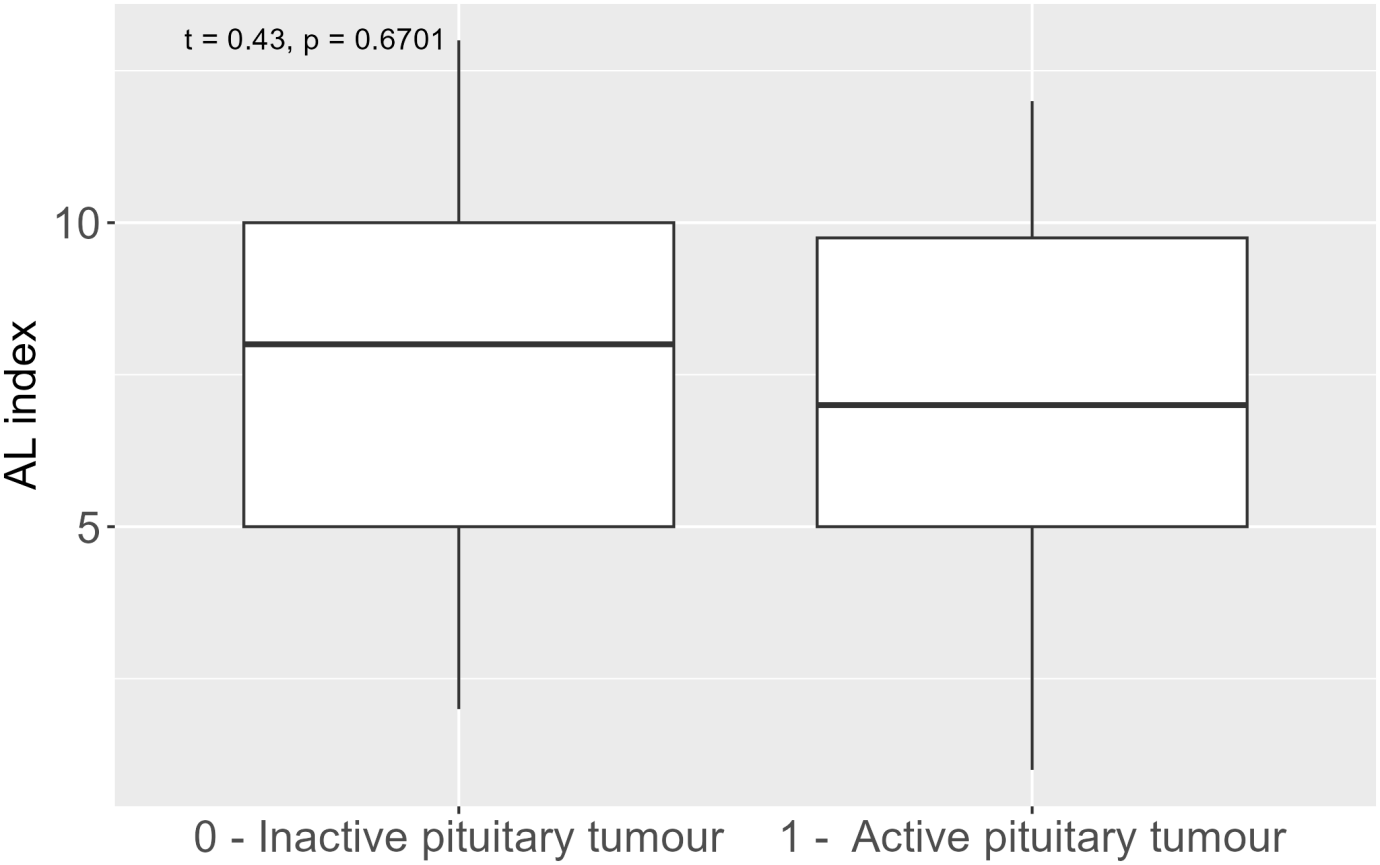
Allostatic load index in inactive and active pituitary tumour.

We found no significant differences in the comparison of total AL index and AL index components between patients with active and inactive pituitary tumour endocrine function (**Figure 3**).

The differences between the groups in the levels of specific biomarkers used to calculate the AL index are shown in **Table 3**. BMI values were significantly higher in the study group compared to the control group (p < 0.001). The mean BMI in patients with pituitary tumours was 29.36, falling within the overweight range, while in the control group it reached 24.22, corresponding to normal. In terms of blood pressure, significant differences were noted between the groups. Both SBP and DBP were higher in the study group (p < 0.001). Patients with pituitary tumours had significantly higher fasting insulin levels (p = 0.004), and in the case of HbA1c, the result was close to the threshold of significance (p = 0.074). Regarding the lipid profile, the study group showed significantly lower HDL cholesterol levels (p = 0.003) and higher triglycerides levels (p = 0.04) compared to the control group. The results of the analysis indicated significantly lower levels of DHEA-S (p = 0.007) and albumin (p < 0.001), and higher levels of IL-6 (p < 0.001) in patients with pituitary tumours, relative to the healthy controls.

**Table 3:** Biomarkers included to calculate the AL index.

Category	Parameter	Sample size (Study group; control group)	Study group	Control group	Test value	p-value
Anthropometrics	BMI, kg/m²	58; 52	29.36 ± 5.52	24.22 ± 3.06	6.12	**< 0.001** ^w^
Cardiovascular	SBP, mmHg	58; 52	134.0 (125.50–141.00)	120.0 (115.00–130.00)	2354.00	**< 0.001** ^m^
	DBP, mmHg	58; 52	82.5 (79.00–90.75)	78.0 (70.00–80.00)	2178.00	**< 0.001** ^m^
	HR, bpm	58; 52	72.0 (65.25–78.75)	72.0 (70.00–80.00)	1373.50	0.419^m^
Carbohydrate metabolism	Glucose, mg/dL	57; 52	89.00 (83.00–99.00)	88.50 (85.00–93.25)	1604.50	0.459^m^
	Insulin, µU/mL	55; 50	7.87 (3.56–13.40)	5.47 (2.33–8.38)	1818.50	**0.004** ^m^
	HbA1c, %	58; 52	5.55 (5.30–5.90)	5.40 (5.18–5.60)	1806.00	0.074^m^
Lipid profile	TC, mg/dL	58; 52	177.74 ± 37.19	186.50 ± 36.51	−1.25	0.216^w^
	LDL, mg/dL	58; 52	101.10 ± 33.04	108.40 ± 29.50	−1.22	0.224^w^
	HDL, mg/dL	58; 52	53.47 ± 11.03	61.33 ± 15.47	−3.04	**0.003** ^w^
	TG, mg/dL	58; 52	96.5 (68.50–141.75)	75.5 (56.00–93.25)	1052.00	**0.004** ^m^
Neuroendocrine	Cortisol, µg/dL	58; 51	10.10 (8.00–13.50)	9.20 (8.35–11.15)	1578.00	0.550^m^
	DHEA-S, µg/dL	58; 52	98.55 (30.75–184.25)	157.50 (99.10–256.50)	1057.50	**0.007** ^m^
Inflammatory markers	CRP, mg/L	58; 52	0.85 (0.50–2.25)	0.80 (0.50–1.43)	1545	0.823^m^
	IL-6, pg/mL	58; 52	3.79 (2.56–6.92)	1.87 (1.57–2.55)	2403	**< 0.001** ^m^
	Albumin, g/dL	58; 52	4.41 ± 0.27	4.76 ± 0.25	−7.13	**< 0.001** ^w^

Study group, control group, ^W^—Welch’s t-test; ^m^—Mann–Whitney test; x ± SD/Me (Q1–Q3); x ± SD—mean and standard deviation; Me (Q1–Q3)—median and quartiles; *p*—statistical significance. BMI, body mass index; SBP, systolic blood pressure; DBP, diastolic blood pressure; HR, heart rate; HbA1c, glycated haemoglobin; TC, total cholesterol; LDL, low-density lipoprotein cholesterol; HDL, high-density lipoprotein cholesterol; TG, triglycerides; DHEA-S, dehydroepiandrosterone sulphate; CRP, C-reactive protein; IL-6, interleukin 6. Bold values - statistical significance, p < 0.05

A comparison of selected metabolic and hormonal parameters in the study and control groups is presented in **Tables 4**, **5**. The study group was characterised by significantly more unfavourable indicators of atherogenicity: higher AIP index (p < 0.001) and higher Castelli index 1 (p < 0.001) and insulin resistance: higher HOMA-IR (p = 0.008) and lower QUICKI (p = 0.008), compared to the control group. In addition, patients with pituitary tumours reported significantly higher uric acid concentrations (p = 0.003). Significantly higher levels of IGF-I (p < 0.001), IGFBP-3 (p = 0.003), and ACTH (p < 0.001) were shown in the study group. Lower concentrations of TSH (p = 0.022), fT3 (p < 0.001), SHBG (p = 0.021) were found in patients with pituitary tumours compared to the control group.

**Table 4:** Metabolic parameters in the group of patients with pituitary tumours and in the control group.

Parameter	Sample size (Study group; control group)	Study group	Control group	Test value	p-value
Castelli index 1	58; 52	1.81 (1.25–2.87)	1.20 (0.86–1.68)	2067.00	**< 0.001** ^m^
Castelli index 2	58; 52	1.86 (1.37–2.53)	1.69 (1.39–2.22)	1629.50	0.469^m^
API	58; 52	−0.08 ± 0.27	−0.26 ± 0.24	3.84	**< 0.001** ^w^
AC	58; 52	2.33 (1.63–3.06)	1.96 (1.61–2.55)	1763.00	0.128^m^
HOMA-IR	55; 50	1.52 (0.79–3.12)	1.21 (0.48–1.83)	1786.00	**0.008** ^m^
QUICKI	55; 50	0.36 (0.32–0.40)	0.37 (0.35–0.44)	964.00	**0.008** ^m^
Uric acid, mg/dL	43; 51	5.51 ± 1.74	4.59 ± 0.99	3.06	**0.003** ^w^
Haemoglobin, g/dL	58; 52	13.30 (12.70–14.07)	13.85 (12.88–15.03)	1219.50	0.085^m^
Creatinine, mg/dL	58; 52	0.75 (0.69–0.88)	0.80 (0.71–0.89)	1318.00	0.256^m^
eGFR, ml/min/1.73m²	58; 52	95.00 (81.00–111.75)	93.00 (81.50–101.50)	1695.50	0.263^m^
Vitamin D, ng/mL	56; 50	33.45 (24.92–43.80)	34.40 (26.92–44.68)	1425.00	0.877^m^
NT-proBNP, pg/mL	35; 43	57.00 (25.70–102.00)	48.80 (28.35–83.90)	772.00	0.848^m^

Study group, control group, ^W^—Welch’s t-test; ^m^—Mann–Whitney test; x ± SD/Me (Q1–Q3); x ± SD—mean and standard deviation; Me (Q1–Q3)—median and quartiles; *p*—statistical significance. API, plasma atherogenic index; AC, atherogenic coefficient; HOMA-IR, homeostatic model assessment of insulin resistance; QUICKI, quantitative insulin sensitivity check index; eGFR, estimated glomerular filtration rate; NT-proBNP, N-terminal prohormone of brain natriuretic peptide. Bold values - statistical significance, p < 0.05

**Table 5:** Hormonal parameters in the study group and the control group.

Hormones	Sample size (Study group; control group)	Study group	Control group	Test value	p-value
IGF-I x ULN	57; 52	0.70 (0.46–0.96)	0.52 (0.46–0.58)	2060.00	**< 0.001** ^m^
GH, ng/mL	53; 50	0.70 (0.36–3.78)	0.64 (0.15–1.83)	1558.50	0.124^m^
IGFBP-3, µg/mL	49; 50	6.08 (4.76–7.34)	5.11 (4.62–5.76)	1652.50	**0.003** ^m^
TSH, mIU/L	57; 52	0.78 (0.62–1.52)	1.17 (0.96–1.57)	1652.50	**0.022** ^m^
fT3, pmol/L	55; 50	4.28 ± 0.53	4.69 ± 0.51	−4.04	**< 0.001** ^w^
fT4, pmol/L	58; 52	13.07 ± 1.89	12.75 ± 1.32	1.05	0.297^w^
ACTH, pg/mL	56; 51	22.30 (12.60–32.18)	12.40 (9.68–17.30)	1989.50	**< 0.001** ^m^
FSH, IU/L	57; 52	5.88 (4.12–13.70)	5.86 (4.14–14.47)	1445.00	0.825^m^
LH, IU/L	55; 52	4.42 (2.54–8.87)	4.28 (2.97–16.77)	1267.50	0.313^m^
Estradiol, pg/mL	48; 41	22.60 (20.00–46.82)	26.90 (20.00–56.80)	924.50	0.613^m^
Testosterone, nmol/L	53; 44	1.42 (0.69–8.54)	1.36 (0.69–12.98)	1039.00	0.351^m^
SHBG, nmol/L	51; 50	37.20 (20.80–53.00)	46.15 (33.02–64.83)	935.50	**0.021** ^m^
FAI, %	50; 44	4.51 (1.80–31.99)	3.28 (1.17–46.23)	1151.00	0.702^m^
PRL, ng/mL	57; 52	8.91 (6.10–15.40)	7.72 (5.77–10.04)	1777.50	0.074^m^

Study group, control group, ^W^—Welch’s t-test; ^m^—Mann–Whitney test; x ± SD/Me (Q1-Q3); x ± SD—mean and standard deviation; Me (Q1–Q3)—median and quartiles; *p*—statistical significance. IGF-I, insulin-like growth factor 1; ULN, upper limit of normal; GH, growth hormone; IGFBP-3, insulin-like growth factor binding protein; TSH, thyroid-stimulating hormone; fT3, free triiodothyronine; fT4, free thyroxine; ACTH, adrenocorticotropic hormone; FSH, follicle-stimulating hormone; LH, luteinising hormone; SHBG, sex hormone binding globulin; FAI, free androgen index; PRL, prolactin.

Bold values - statistical significance, p < 0.05.

## 4 Discussion

Our study demonstrated that patients with pituitary tumours present a significantly elevated allostatic load (AL) index compared with age- and sex-matched controls, regardless of adenoma hormonal activity. This finding suggests that pituitary disease, independent of endocrine status, contributes to cumulative multisystem stress.

Pituitary adenomas (PitNET) are common, occurring in 10% of the population, but the vast majority remain harmless throughout life. They account for about 15% of all primary brain tumours, making them the third most common of all brain tumours (31, 32). Adenomas differentially affect patient morbidity and mortality depending on cell type, hormone secretion activity and growth behaviour. The endocrine activity of pituitary tumours, whether acromegaly, Cushing’s disease, prolactinoma or TSH-oma, causes metabolic and endocrine dysfunction and affects the hypothalamic-pituitary-adrenal (HPA) axis. The body adapts to the altered adverse conditions and seeks to maintain allostasis. Prolonged exposure to pathogenic factors leads to multisystem wear and tear and adverse changes resulting in the development of allostatic load (1). Regular assessment of allostatic load may allow for long-term monitoring of the patient and understanding the reasons why the patient continues to have symptoms despite effective treatment of pituitary tumors, which is often observed in clinical practice. Taking allostatic load into account can help distinguish significant clinical differences between patients who otherwise appear deceptively similar because they share the same medical diagnosis. To improve therapeutic effectiveness, it is necessary to shift from a purely biomedical approach to a psychosomatic approach that takes into account quality of life.

In our study, patients with pituitary tumours were significantly more frequently affected by chronic diseases (nicotinism, hypertension, insulin resistance, pre-diabetes and diabetes) compared to the control group. As emphasized in the literature, acromegaly, which was most common in patients in our study group, is associated with a significantly increased risk of cardiovascular complications, including hypertension, arrhythmia, and acromegalic cardiomyopathy, which are major factors affecting morbidity and mortality (33). Excessive exposure to cortisol in Cushing’s disease also determines an increased incidence of cardiovascular and metabolic diseases, which reduce survival and are the main cause of death (34).Vaccarino et al. explain the association of the accumulation of a long-term external factor—stress—on the development of chronic diseases, particularly cardiovascular disease (35). Constant increases in stress mediators (including epinephrine, glucocorticoids, cytokines) can cause dysregulation of several major systems (including the sympathetic-adrenal system, the HPA axis, and the cardiovascular, metabolic, nervous, endocrine and immune systems), tissue damage or desensitisation of receptors (36, 37). Inhibited immune function (by glucocorticoids), atherosclerosis and obesity (by cytokines), and anxiety and depression showing atrophy of nerve cells in the brain (by cortisol) may be examples of chronic diseases associated with allostatic stress (38).

In the literature, there are studies of the AL index in patients with other endocrine diseases, but there is a lack of studies assessing patients with pituitary tumours, which makes our analysis novel. The relatively simple and easily accessible model for calculating the AL index is a promising tool useful in daily clinical practice.

Sonino et al. indicate a higher AL index in patients with the active phase of primary hyperaldosteronism compared to the inactive form and to people with primary hypertension (39). Moreover, the same author in another study shows that patients with pituitary disease had significantly higher levels of allostatic load in the PsychoSocial Index than healthy controls but not compared to patients with endocrine disease not related to the pituitary gland (40). This suggests similar levels of AL in different types of endocrine disorders and points to the need for AL index studies among different endocrinopathies.

In Graves-Basedow disease and hyperprolactinemia (with an aetiology of prolactin-secreting pituitary tumours and in idiopathic form), the influence of stressful life events on the pathophysiology of the disease in question has been demonstrated (41, 42). In addition, a study by Sonino et al. found that stress contributed to the development of Cushing’s syndrome of pituitary aetiology, while it did not affect the Cushing’s syndrome of independent pituitary origin (primary adrenal hyperfunction and ectopic ACTH production), which supports the hypothesis of the involvement of the limbic-hypothalamic system in the pathogenesis of this condition (43). These reports demonstrate the importance of allostatic load and the study of clinimetric data in endocrine diseases. The next phase of our study will be to assess clinimetric tools, including an analysis of psychological and psychiatric variables, to completely evaluate the allostatic load in a group of patients with pituitary tumours.

A significant limitation of our study is the small sample size and the predominance of patients with acromegaly in this group. Therefore it is advisable to continue the analysis on a larger number of patients to assess the usefulness of the AL index as a marker for the early detection of cumulative health risks in patients with pituitary adenoma as well as any others hormonal disorders. It is also reasonable to extend the research to other centers in Poland and worldwide, which could assess the impact of different genetic backgrounds and lifestyles of the studied population. Moreover, it is worth considering the analysis of the AL index in individual pituitary diseases, as each hormonal activity of a pituitary adenoma is subject to various pathophysiological processes.

The calculation of the Allostatic Load (AL) index in this study was based on the classic framework proposed by Seeman et al. (30), which incorporates biomarkers representing six major stress-related physiological systems. But due to the complex and multisystem nature of allostatic load, there is a lack of a consistent definition and standardized markers for calculating this index. It’s important to note that different allostatic load studies often utilize different sets of biomarkers. In our study, the analysis of catecholamines was not feasible due to logistical constraints. The planned 24-hour urine collection required for catecholamine analysis was impossible, as the participants were hospitalized for only a short duration. Moreover, performing blood analysis for catecholamines was also not an option due to the limited time window available to process the blood samples, which is critical for accurate measurements. These differences in marker selection further complicate comparisons between studies. The results of the meta-analysis by McCrory et al. indicate that the AL index, calculated based on five biomarkers [CRP, resting heart rate (RHR), HDL cholesterol, waist-to-height ratio (WtHR), and HbA1c], predicted independent mortality outcomes as effectively or better than more complex biomarker sets (44). Further research is needed to standardize the formulas for calculating the AL index and this could improve both the comparability and reliability of allostatic load assessments across different contexts.

Given the limited number of study participants and the presence of prevalent chronic conditions in the control group (e.g., hypertension and hypercholesterolemia), the present study employed a norm-based point assignment approach for the calculation of the allostatic load (AL) index. This methodologyoffers the advantage of prioritizing clinical significance over population-based distribution, thereby facilitating a more straightforward interpretation of results, particularly in instances where reference norms are well established. The use of this method was also recommended by a statistician and has been previously used in other studies (45, 46). A limitation of this approach, however, lies in its inability to convey the magnitude by which individual biomarkers deviate from normative ranges. Accordingly, in the absence of clearly defined reference standards, the application of this method is not recommended. In analyzing the results of our study, we assigned the maximum number of points in particular categories to participants receiving pharmacological treatment to account for the presence of chronic diseases that impact allostatic load independently of the treatment’s effect. However, we acknowledge that this approach may lead to a systematic overestimation of the AL index, as it does not reflect individual responses to treatment or the degree of biomarker normalization. Therefore, the results should be interpreted with consideration of this potential bias. A similar approach regarding pharmacotherapy was applied in the study by Waliszewska−Prosół et al. (18).

Our study showed that age was a significant predictor of higher total AL index and individual metabolic components, regardless of whether the pituitary tumour was hormonally active or inactive. This suggests an age-related deterioration of physiological regulatory mechanisms, leading to an accumulation of biological stress over time. In the study by Seeman et al., which analysed the AL index in 70–79-year-olds, higher baseline AL index results were associated with a significantly increased risk of 7-year mortality, as well as impaired cognitive and physical functioning. Furthermore, these scores were marginally correlated with cardiovascular incidents, independent of standard socio-demographic characteristics and baseline health status (30). These findings support the concept of AL as a measure of cumulative biological load, especially in an elderly population with multiple chronic comorbidities. This variety of diseases underscores the need for biomarkers that can signal early signs of dysregulation of multiple systems. The study of Volarić et al. showed a decrease in the physiological and psychological variance of some AL biomarkers with age. Allostatic biomarkers, which show significant variability in older adults (cortisol to DHEA ratio, adrenaline, noradrenaline, IL-6, CRP, fibrinogen, HDL cholesterol, creatinine, and systolic and diastolic blood pressure), are best used to assess responses to external stress. However, BMI and IL-6 are two parameters that mostly indicate deterioration in health in older, generally healthy individuals (47). Most of these biomarkers were examined in our study.

In our analysis, education was not a significant prognostic factor of AL index level or its subscales in any of the models. The absence of an observed association between education and AL index may reflect the relatively homogeneous study population. Limited variability in socioeconomic characteristics could reduce the ability to detect relationships, and recruitment from similar backgrounds may introduce selection bias. Therefore, these findings may not generalize to more diverse populations. It would be beneficial to perform the analysis with different education levels in future studies. Contrary to our analysis, in the results of the cohort study by Li et al., an association between education level and allostatic load was found. In the US population, men with lower education levels and high AL index had a fourfold increased risk of cancer mortality (48). These data suggest that lack of a secondary education may be associated with limited economic opportunities, leading to poverty and poorer access to healthcare services. Population-based studies indicate that allostatic load also increases due to unfavourable conditions, such as low socioeconomic status, living in poor neighbourhoods, lower education levels, ethnicity and racial discrimination (49). Therefore, it is valuable to expand the study by analysing clinimetric data that considers the patient’s sociopsychological profile.

Comparing the components of the AL index, we observed a predominance of lipid metabolism parameters in both the study and control groups, which may suggest the frequent occurrence of dyslipidemia from both a potentially healthy population—the control group—and in patients with pituitary tumours. In the results of the WOBASZ II study, assessing the prevalence of components of the metabolic syndrome meeting the 2022 criteria, conducted on a sample of 6170 adult Poles, atherogenic dyslipidemia was noted in 67.6% of the subjects, which classifies this disease as the most common component in the study population (50). Similar to our analysis, in the American population study, higher AL, reflecting cumulative physiological stress, was significantly associated with abnormal lipid profiles, particularly increased LDL and triglyceride levels and decreased HDL levels (51).

The other predominant AL index components in the study group assessed other components of the metabolic syndrome, while the control group had neuroendocrine and inflammatory components. The differences in the structure of the dominant components suggest that there is more advanced dysregulation of physiological systems in the study group, especially those related to metabolism, while the activity of these systems remains within the limits of relative equilibrium in the control group, with minor inflammatory-hormonal changes, which may reflect a background of subclinical physiological load in the healthy population as well.

The neuroendocrine component of the AL index, which is an indirect measure of inflammatory-hormonal activation, was found to be significantly higher in patients with the disease (both in the entire study group and in the subgroup with acromegaly) than in healthy subjects when IGF-I levels were included in the calculations. Especially in the context of patients with acromegaly, in whom we observed a stronger difference in the neuroendocrine component of the AL index relative to the control group, a chronic excess of growth hormone and IGF-I promotes chronic inflammation and metabolic dysfunction. Analysis of the AL index should be considered as an additional biomarker to support the diagnosis and monitoring of patients with pituitary tumours, especially patients with acromegaly.

In our analysis, BMI proved to be the dominant anthropometric parameter differentiating the groups. The study group presented an increased risk of overweight and obesity. This indicates the need for weight control among endocrine patients, including those with pituitary dysfunction, especially since BMI is a simple diagnostic indicator.

A retrospective study by Andrzejak et al. assessing the association between AL index and cancer mortality according to BMI status also demonstrated a significant role of increased BMI in the induction of inflammation and metabolic disorders. They found an increased risk of cancer mortality by 3%, 31%, and 39% in participants who were underweight and normal weight (BMI < 24.9 kg/m²), overweight (BMI 25–29.9 kg/m²), and obese (BMI > 30.0 kg/m²) with high AL compared to low AL (52). The study by Prunell-Castañé et al. also indicates inflammatory and cardiometabolic complications associated with increased body weight in the younger population. A correlation between poorer cognitive function and higher AL scores, but only in overweight/obese adolescents and young adults was found (53).Moreover, among the biomarkers needed to calculate the AL index, we observed higher values of SBP and DBP in the study group compared to the control group. In addition, in our study, we noted higher fasting insulin levels and triglycerides levels and lower HDL cholesterol levels in the group of patients with pituitary tumours. Among other metabolic parameters, the analysis revealed higher uric acid levels and higher values of atherogenicity and insulin resistance indices in the study group. The above results may indicate an increased cardiometabolic risk in patients with pituitary tumours.

Literature data confirm that in acromegaly, the most prevalent condition in our study group, GH and IGF-I contribute to the development of cardiovascular and metabolic complications through systemic inflammation, endothelial dysfunction, and insulin resistance (54).

Lower DHEA-S levels, characteristic of patients from our study group, may suggest impaired activity of the hypothalamic-pituitary-adrenal axis (HPA) and greater exposure to chronic stress. This hormone has an antagonistic effect on glucocorticosteroids inhibits the synthesis and secretion of catecholamines (22). In addition, DHEA-S exhibits neuroprotective (protects the hippocampus from the neurotoxic effects of corticosterone) and anti-inflammatory effects [decreases the level of NF-κВ, interleukin-1β (IL-1β), tumour necrosis factor-α (TNF-α) and interferon-γ (IFN-γ)] (36, 55). Low levels of DHEA-S and age-related decline in this hormone may result in higher levels of circulating cortisol in peripheral target tissues, contributing to insulin resistance, obesity and the metabolic syndrome (through increased gluconeogenesis, increased free fatty acids) (56). In patients with pituitary tumours, we also found higher levels of IL-6 and lower levels of albumin. The above constellation of results is associated with increased activity of chronic inflammatory processes, which are a consequence of long-term stress (57). Unfavourable differences in anthropometric, cardiovascular, metabolic, neuroendocrine and inflammatory parameters in patients from the study group indicate chronic exposure to environmental and psychosocial stressors that predispose to the development of diseases of civilization.

The observed discrepancy between an elevated AL index and the absence of significant differences in certain individual biomarkers, such as CRP, total cholesterol, and LDL cholesterol, can be explained by several factors. The AL index represents a cumulative measure that integrates multiple physiological systems; thus, even in the absence of differences in selected biomarkers, cumulative dysregulation across systems may result in a significantly higher overall score. Additionally, compensatory mechanisms may contribute to maintaining certain biomarkers within the normal range, while dysfunction in other systems drives the elevation of the AL index. Some biomarkers, such as CRP, exhibit considerable intra-individual variability and may be less sensitive to chronic physiological stress compared to composite measures. These considerations underscore the value of the AL index as an integrated diagnostic tool capable of capturing multisystem regulatory disturbances that may not be evident when examining individual biomarkers in isolation.

Our comparative analysis of pituitary and peripheral organ hormone concentrations revealed significant differences across several hormonal axes between the study and control groups. Within the somatotropic axis, higher IGF-I and IGFBP-3 concentrations were observed in the study group. This could suggest increased activity of the GH-IGF-I axis and potentially reflect metabolic adaptation to chronic stress.

Since 41 patients in the study group were diagnosed with acromegaly, we calculated the total AL index after incorporating IGF-I concentrations. We observed significantly higher allostatic loads for both the entire study group and the subgroup of patients with acromegaly compared to the control subjects. These results suggest that patients with pituitary tumors, particularly somatotropinomas, experience long-term activation of compensatory mechanisms responsible for adaptation to stress, which may lead to physiological deterioration over time. The inclusion of IGF-I in the calculation of the AL index represents an innovative methodological approach, as it adds an endocrine dimension to the assessment of multisystem physiological dysregulation. IGF-I as marker was also use in the previous studies (58, 59). However, the interpretation of these results may be complicated by the fact that chronic conditions or metabolic disorders can independently alter IGF-I levels. Moreover, the presence of functioning pituitary adenomas undoubtedly affects the hormonal profile, significantly limiting the ability to interpret the results solely in the context of allostatic mechanisms. Therefore, it is important to acknowledge these limitations and consider their implications when interpreting the findings. Future analyses would benefit from comparing larger patient cohorts, taking into account adenoma type (functioning vs. non-functioning) and the potential presence of pituitary insufficiency, with particular attention to each hormonal axis.

Due to the wide variability of GH levels, this hormone cannot be considered a reliable allostatic marker. Regarding the hypothalamic-pituitary-thyroid (HPT) axis, the study group showed reduced levels of TSH and free T3 (fT3), a hormonal pattern consistent with the low T3 syndrome, which is frequently observed in chronic stress or disease states (60). In the hypothalamic-pituitary-adrenal (HPA) axis, we observed elevated ACTH concentrations accompanied by reduced DHEA-S levels, with cortisol levels remaining unchanged. This pattern may reflect either a partial impairment or dysregulation of the HPA axis in the context of chronic stress. As for gonadal hormones, no significant group differences were noted. However, the lower SHBG levels in the study group may suggest altered bioavailability of free sex hormones. The prolactin levels were also higher in the study group, and while the difference approached statistical significance, it may reflect an adaptive neuroendocrine response to prolonged stress exposure. Overall, these results confirm the neuroendocrine pathophysiology associated with chronic stress in patients with pituitary diseases.

## 5 Conclusions

The findings of this study support the utility of the AL index as a comprehensive measure of cumulative physiological stress in patients with pituitary disorders. Observed differences in AL components may have important clinical implications for the prevention, monitoring, and early intervention of associated complications, encompassing anthropometric, metabolic, neuroendocrine, and inflammatory parameters. The relatively small sample size represents a key limitation, underscoring the preliminary nature of these results and the need for further research in larger and more heterogeneous cohorts. Future studies should consider adenoma subtype and pituitary function, incorporate additional biomarkers of allostatic load, and employ longitudinal designs to capture its temporal dynamics, particularly in ageing populations. Future research should aim to standardize methods for measuring AL and investigate the long-term effects of chronic allostatic load on health. Continued development and validation of the AL index across different populations and disease entities is recommended.

## Data Availability

The raw data supporting the conclusions of this article will be made available by the authors, without undue reservation.
