# Supplementary material for: Allostatic load index in patients with pituitary tumours: a case control study
Source: Front Endocrinol (Lausanne). 2025 Oct 29;16:1676246. doi: 10.3389/fendo.2025.1676246 (PMC12605342; doi:10.3389/fendo.2025.1676246)
Supplement: Supplementary file 1 [file Table1.docx]

Supplementary Material

# Supplementary Tables

**Table S1:** Shapiro-Wilk normality tests in the study group.

| Parameters | Study group | | |
| --- | --- | --- | --- |
|  | Sample size | W | p |
| Age, years | 58 | 0.971 | 0.179 |
| Weight, kg | 58 | 0.933 | 0.003 |
| Height, cm | 58 | 0.972 | 0.199 |
| BMI, kg/m² | 58 | 0.478 | 0.000 |
| HR, bpm | 58 | 0.113 | 0.000 |
| SBP, mmHg | 58 | 0.582 | 0.000 |
| DBP, mmHg | 58 | 0.630 | 0.000 |
| Illness duration, years | 58 | 0.780 | 0.000 |
| Max tumor size, cm | 44 | 0.892 | 0.001 |
| Fat, % | 21 | 0.951 | 0.350 |

**Table S2:** Shapiro-Wilk normality tests in the control group.

| Parameters | Control group | | |
| --- | --- | --- | --- |
|  | Sample size | W | p |
| Age, years | 52 | 0.964 | 0.121 |
| Weight, kg | 52 | 0.974 | 0.310 |
| Height, cm | 52 | 0.971 | 0.235 |
| BMI, kg/m² | 52 | 0.632 | 0.000 |
| HR, bpm | 52 |  |  |
| SBP, mmHg | 52 | 0.554 | 0.000 |
| DBP, mmHg | 52 | 0.372 | 0.000 |
| Illness duration, years | - |  |  |
| Max tumor size, cm | - |  |  |
| Fat, % | 27 | 0.953 | 0.260 |

**Table S3:** The comparison of model goodness-of-fit (AIC) and the model distinguishability test (Vuong test – non nested likelihood ratio test). The results of the hypothesis: ZIP model is better than Poisson model is presented.

| AL index | AIC | | Vuong test | | |
| --- | --- | --- | --- | --- | --- |
|  | ZIP | Poisson | z | | p |
| Cardiovascular | 274.8 | 272.8 | -0.097 | 0.539 | |
| Carbohydrate metabolism | 197.2 | 201.8 | 1.336 | 0.091 | |
| Lipid profile | 359.7 | 363.3 | 1.217 | 0.112 | |
| Neuroendocrine | 232.3 | 230.3 | -0.951 | 0.829 | |
| Inflammatory markers | 236.8 | 234.8 | -0.961 | 0.832 | |

Explanation: AIC – Akaike Information Criterion, ZIP – zero-inflated Poisson regression, Poisson – Poisson regression, z – test value, p – statistical significance
